# Correction to: Epidemiological and clinical characteristics of patients with suspected COVID-19 admitted in Metro Manila, Philippines

**DOI:** 10.1186/s41182-021-00373-5

**Published:** 2021-10-22

**Authors:** Eumelia P. Salva, Jose Benito Villarama, Edmundo B. Lopez, Ana Ria Sayo, Annavi Marie G. Villanueva, Tansy Edwards, Su Myat Han, Shuichi Suzuki, Xerxes Seposo, Koya Ariyoshi, Chris Smith

**Affiliations:** 1 San Lazaro Hospital, Manila, Philippines; 2 School of Tropical Medicine and Global Health, Nagasaki University, Nagasaki, Japan; 3 MRC Tropical Epidemiology Group, London School of Hygiene and Tropical Medicine, London, UK; 4 Institute of Tropical Medicine, Nagasaki University, Nagasaki, 852-8523 Japan; 5 Faculty of Infectious and Tropical Diseases, London School of Hygiene and Tropical Medicine, London, UK

## Correction to: Trop Med Health (2020) 48:51 https://doi.org/10.1186/s41182-020-00241-8

Following the publication of the original article [1], some content was missing in both the second paragraph of the section Study design and participants and the section Ethics approval and consent to participate.

The updated sentence is given below and the changes have been highlighted in **bold typeface**.

This was a retrospective analysis of anonymized routinely collected data, **implemented under an existing acute respiratory tract/COVID-19 study**, approved by the SLH research ethics and review unit (Ref: SLH-RERU-2020-022-I) and the School of Tropical Medicine and Global Health, Nagasaki University Ethical Committee (NU_TMGH_2020_119_1).

The original article [1] has been corrected.
